# WHAT ROLE DO SOCIAL RELATIONSHIPS PLAY IN HURRICANE SURVIVAL?

**DOI:** 10.1093/geroni/igad104.1589

**Published:** 2023-12-21

**Authors:** Hope Mimbs, Anne Barrett, Jessica Noblitt, Cherish Michael, Brianna Soulie

**Affiliations:** Florida State University, Tallahassee, Florida, United States; Florida State University, Tallahassee, Florida, United States; Florida State University, Tallahassee, Florida, United States; Florida State University, Tallahassee, Florida, United States; Florida State University, Tallahassee, Florida, United States

## Abstract

Despite the disproportionately high hurricane mortality rates for older adults, we know little about how hurricane preparedness and evacuation decisions are influenced by social relationships, specifically interaction with family and friends. Using data from an online survey of Floridians aged 50 and older that was conducted between December 2020 and April 2021 (n=3,797), we examine the association between frequency of interaction with family and friends and two hurricane survival measures: hurricane preparedness and likelihood of evacuation. We found that interacting more frequently with friends predicts greater hurricane preparedness, as well as a greater likelihood of evacuation. We also found that interacting more frequently with family members predicts a greater likelihood of evacuation. Our study highlights yet another benefit of social relationships: Interacting more often with friends and family can save lives during emergencies.

